# COMVC-19, a Program to Protect Healthcare Workers’ Mental Health During the COVID-19 Pandemic, and the Second Wave of the Pandemic: A New Moment and the Impact of Previous Experiences

**DOI:** 10.6061/clinics/2021/e3574

**Published:** 2021-11-23

**Authors:** Fabio Cassiodoro Veiga Scarduelli, Pedro Fukuti, Felipe Corchs, Euripedes Constantino Miguel, Eduardo de Castro Humes

**Affiliations:** I Hospital das Clinicas HCFMUSP, Faculdade de Medicina, Universidade de Sao Paulo, Sao Paulo, SP, BR.; II Departamento de Psiquiatria, Faculdade de Medicina FMUSP, Universidade de Sao Paulo, Sao Paulo, SP, BR.; III Grupo de Assistencia Psicologica ao Aluno, Faculdade de Medicina FMUSP, Universidade de Sao Paulo, Sao Paulo, SP, BR

Dear Editor,

We would like to further elaborate on and discuss the experiences presented in Fukuti et al.’s paper titled, “COMVC-19: A Program to protect healthcare workers' mental health during the COVID-19 Pandemic. What we have learned,” published in “Clinics” (1). The original article provided information on the development and implementation of a program to assist healthcare workers (HCWs) facing increased mental health demands during the first wave of the pandemic. These HCWs served at Brazil’s largest public hospital, the Hospital das Clínicas da Faculdade de Medicina da Universidade de São Paulo (HCFMUSP). In this article we presented our initiative, the COMVC-19 program. The name is an attempt to play with the Portuguese expression with (com) you (você, informally shortened to “vc”) and the COVID-19 pandemics, and the full name of the program was “COMVC19: The Mental Health and Psychosocial Well-Being Personal Protective Equipment to the Health Professionals involved in the Combat against the COVID-19 Pandemic”.

As of September 2021, there have been over 200 million confirmed cases of COVID-19 and 4 million deaths worldwide, despite proactive vaccination efforts (2). The COVID-19 pandemic has demanded radical changes in the healthcare delivery protocols employed by healthcare institutions and their HCWs. In March 2020, most people thought that the measures adopted to combat the pandemic would be needed for just a few months. However, today, healthcare institutions worldwide are ready for the possibility of a third wave of cases majorly related to the new variants of the virus.

During the first wave of infections, we learned that the increasing stress associated with the COVID-19 pandemic had taken a great toll on the mental health of HCWs. To elaborate, when surveyed about their greatest sources of stress associated with the pandemic, HCWs cited work overload, infection risks, and the constant and unpredictable redeployment to novel medical roles as particularly stressful. The widespread movement restrictions, constant work performance evaluations, and concerns about their own well-being as well as that of their loved ones, only added to their stress. Indeed, there was a significant increase in cases of burnout, anxiety, and depression among HCWs during the pandemic (3).

Following the first spike in cases, subsequent wave patterns began to occur in multiple countries, including Brazil. In our country, the second wave was particularly challenging as the patients were younger and presented with an increased need for ventilation support (4). By the last week of March 2021 (the 14^th^ epidemiological week), there were over 400,000 confirmed cases and 20,000 deaths per week (2). The second wave prompted a diverse array of psychological reactions, including rage, fear, and feelings of hopelessness and ineffectiveness, in both HCWs and the general population.

The HCFMUSP responded to the pandemic by reallocating its resources to focus on COVID-19 patients during the most demanding periods of the pandemic, namely, the first and second waves. During the first wave, the COMVC-19 program provided mental health assistance to the HCWs, including a 24/7 hotline, supportive psychotherapy, and telepsychiatry consultations. While the symptoms of distress remitted in most of the HCWs and they were soon discharged, some HCWs required referral to non-temporary outpatient services. The numbers of HCWs seeking help dwindled by August 2020, and thus, the COMVC-19 program was paused. Our experience with the first wave of COVID-19 showed that surge-related mental health assistance provision for HCWs, is viable and efficacious.

In March 2021, following the emergence of the second wave of COVID-19 in Brazil, the Mental Health Assistance branch of the COMVC-19 in the HCFMUSP was reinstated (Table 1). Given what we had learned during the first wave of the pandemic, we implemented certain changes in the program. For example, the hotline system was decentralized; each institute's psychology department was responsible for the screening and the provision of psychological support of their own HCWs. Furthermore, a minority of the HCWs were screened by psychiatry residents to promote faster support and screening. During these screenings, the HCWs were referred either to brief supportive psychotherapy sessions (with either the institute's psychologists or a group of voluntary psychotherapists), or to psychiatric consultations with the psychiatry residents (decided on a case-by-case basis). Following each screening interview or psychiatric consultation of the HCWs, a healthcare professional documented the sociodemographic data, clinical impressions, and (for the psychiatrists) diagnostic implications based on the International Classification of Diseases, Tenth Revision (ICD-10). The data collection methods used were the same as those of the previous publication (1), for which ethics committee approval was obtained.

Of the 166 HCWs screened from April to September 2021, most were women (86.7%), with a mean age of 37 years. HCWs were most frequently members of the nursing staff (38.0%) (Table 2). Most HCWs (61.4%) had no past psychiatric problems. Of all HCWs that were screened, 86 (51.8%) were referred to brief psychotherapy sessions, and 68 (41.0%) were referred for psychiatric consultation. The most common complaints motivating the referral to psychiatric consultation were anxiety (63.5%) and depressive mood (44.2%). The most frequent primary diagnoses established after the clinical evaluation included major depressive disorder (34.6%), generalized anxiety disorder (21.2%), and adjustment disorder (11.5%).

Contrary to the findings of the COMVC-19 program during the first wave, there was a notable reduction in the prevalence of physicians seeking mental health support (from 22.7% to 3.6%) (1). In addition, there was a major change in the redeployment of doctors between the first and second waves. The main portion of the physician workforce, the medical residents, maintained most of their usual rotations while being redeployed to COVID-19 wards. This difference in rotation structure may have offered a protective effect on the physicians’ mental health.

Another important distinction between the waves was the relative proportion of the diagnoses. There was a decrease in the proportion of adjustment disorders (23.7% to 11.5%) at the expense of depressive disorders (22.1% to 34.6%) (1). At the emergence of the pandemic, during the initial redeployments and restrictions, HCWs intense depressive and anxious reactions could be clearly attributed to the severe impact of those life-events. However, as the pandemic progressed and the events mounted, the symptoms may have assumed a chronic nature, making it harder to determine the exact causal relationship.

The COMVC-19 program provided timely aid to the HCWs in a moment of crisis. An online survey was sent to approximately 21,600 HCWs of the HCFMUSP, of whom 1,000 responded. Preliminary analysis of the data revealed that institutional support was the primary protective factor against the mental health symptoms, with an inverse correlation between the two (Alves, M. O. C., personal communication). The importance of institutional actions in mental health support for HCWs cannot be overstated.

In our experience, the second wave of the pandemic has had a great impact on HCWs mental health, in a way that is distinguishable from the first wave. We hope our experiences might shed light into the possible mental health impacts of this novel situation. In summary, availability of appropriate support systems to HCWs is paramount, especially in times of great distress.

## Figures and Tables

**Table 1:** Number of HCWs assisted via the COMVC-19 program in the 1^st^ and 2^nd^ waves of the COVID-19 pandemic.

	First Wave (March-August 2020)*	Second Wave (April-September 2021)
Screened patients (N)	395	166
Psychiatric assistance (N)	131	52

* Data from Fukuti, P. et al. (1).

**Table 2:** Characteristics of the HCWs who sought Mental Health care under COMVC-19.

	n	mean	range
Age (in years)	166	37	20-61
	N	n	%
Gender	166		
Female		144	86.7%
Male		22	13.3%
Role	166		
Nursing Staff (Registered and Practical)		63	38.0%
Administrative Personnel		52	31.3%
Physicians		6	3.6%
Nutrition Personnel		11	6.6%
Laboratory technician		12	7.2%
Other		22	13.3%
Previous psychiatric diagnosis*	166		
None		102	61.4%
Depression		40	24.1%
Anxiety		40	24.1%
Bipolar Affective Disorder		5	3.0%
Other		6	3.6%
Referral for treatment*	166		
No internal MH referral		24	14.5%
Brief psychotherapy sessions		86	51.8%
Psychiatric consultation		68	41.0%
Psychiatric complaints that motivated the referral for psychiatric consultation*	52**		
Anxiety		33	63.5%
Paroxysmal anxiety episodes		9	17.3%
Depressive Mood		23	44.2%
Dysphoria		9	17.3%
Sleep problems		19	36.5%
Feeling burned out		8	15.4%
Diagnosis established after the clinical evaluation*	52		
Major Depressive Disorder		18	34.6%
Generalized Anxiety Disorder		11	21.2%
Adjustment Disorder		6	11.5%
Mixed anxiety and depressive disorder		3	5.8%
Panic disorder		3	5.8%
Acute Stress Reaction		2	3.8%
Post-Traumatic Stress Disorder		2	3.8%
Other		7	13.5%

* More than one possible outcome: MH: mental health.

** 16 participants did not attend the psychiatric consultation.
